# Biofluids manipulation methods for liquid biopsy in minimally-invasive assays

**DOI:** 10.1016/j.mex.2022.101759

**Published:** 2022-06-17

**Authors:** Valeria Garzarelli, Francesco Ferrara, Elisabetta Primiceri, Maria Serena Chiriacò

**Affiliations:** a University of Salento, Dept. of Mathematics & Physics E. de Giorgi, Via Arnesano, 73100, Lecce, Italy; b CNR NANOTEC – Institute of Nanotechnology, via per Monteroni, 73100, Lecce, Italy; c STMicroelectronics s.r.l., via per Monteroni, 73100, Lecce, Italy

**Keywords:** Sample handling, Body fluid, Biomarkers, Diagnosis, Screening

## Abstract

The Liquid Biopsy (LB) is an opportunity for non-invasive diagnosis and prognosis of various diseases. To date, it isn't possible to consider that tissue biopsy can represent a pathology entirety. Then, body fluids are rich in a large number and variety of biomarkers and they can provide information about several diseases.

• Recently, other biological fluids, easy to be collected are rising for their significant content of biomarkers and for the possibility to collect and manipulate them without the intervention of medical staff.
• The management of biological fluids requires suitable storage methods. Temperature, storage time and physical stresses due to sample handling can lead to chemical and physical changes that may induce sample degradation and incorrect analysis.
• The reliability of a diagnostic or screening test depends on its sensitivity and specificity. As the liquid biopsy is a 'snapshot' of a pathophysiological condition, it is crucial that its components do not degrade due to the improper handling of the body fluid.

In this review, some handling methods of Saliva, Urine, Stool, Seminal Fluid, Tears and Sweat samples will be described, as well as protocols to facilitate the analysis of metabolites, nucleic acids, proteins and Extracellular Vesicles (EVs) from those unusual body fluids.

## Graphical abstract

**Figure:**
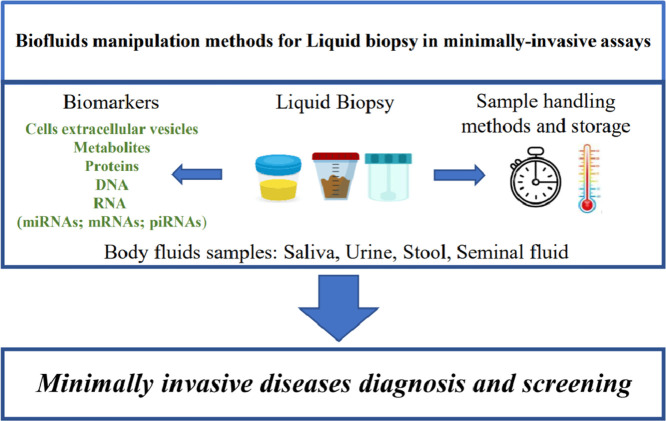

## Specifications table

**Table:** 

Subject Area:	Biochemistry, Genetics and Molecular Biology
More specific subject area:	*Biomarkers; sample handling*
Method name:	*Review paper of methods for biofluids handling (not including blood).*
Name and reference of original method:	*N.A.*
Resource availability:	*N.A.*

## Introduction

60% of the human body is made up of fluids distributed between intracellular and extracellular compartments, the extracellular fluid is divided into Tissue Interstitial Fluid (TIF) and blood plasma [1]; however, as a product of glands or ultra-filtrating blood or as a product of other physiological processes, the human body is able to produce biological fluids such as saliva, urine, faces, seminal fluid, tears and sweat that can provide information about the health state of an individual. As body fluids are a rich source of biomarkers, the Liquid Biopsy (LB) is a chance for non-invasive diagnosis and prognosis of various diseases. In particular, many studies in recent literature have focused on the use of LB to diagnose both easily accessible or hard-localization cancer. It has been shown that tissue biopsy cannot be used to identify the overall picture and evolution of cancer with its metastasis [2]. It is possible, instead, to use body fluids as a replacement for tumor biopsies. LB can be employed to monitor the course of the disease and treatment choices, including the detection of circulating cancer cells, nucleic acids, proteins and other markers. In the last years, as a matter of fact, LB has proved to be a promising minimally invasive diagnostic tool, and it could be a great help in the current COVID-19 pandemic situation, in which cancer patients’ management and follow-up could be challenging [3]. Moreover, thanks to the low volumes of sample usually needed for analysis (few microliters), LB could represent a valid alternative to identify biomarkers of pathologies and repeated analysis are not heavy to bear from patients’ point of view. However, in some cases, some biomarkers could be underestimated due their low concentration. This could be the case of rare circulating cells (Circulating Tumor Cells or rare fetal cells from maternal peripheral blood) or nucleic acid in low-number copy [4], [5]. This condition could take advantage on procedures for sample enrichment in order to identify a valid result of the clinical assay. Also in this case, blood sampling has been referred to as the gold standard, but the other biological fluid have the potential to compensate for these limits and overcome also problems in blood draw [6].

To date, circulating tumor DNA (ctDNA), cell-free DNA (cfDNA), are the only allowed analytes in clinical practice. Also, Circulating Tumor Cells (CTCs), Extracellular Vesicles (EVs), circulating tumor RNA (ctRNA), microRNAs (miRNAs), are emerging target in medicine personalized approach that can help give information on cancer origins and its evolution [7] and that could be corroborated in near future [8]. In particular, EVs are a communication way among cells for the mutual transfer of membranes, cytosolic proteins, lipids and RNA is displayed in body fluids a wide range of roles that can disclose the specifics of cancer [9].

Among the targets of liquid biopsy, ultra-short DNA fragments are considered a potential biomarker of early stages of cancer and screened in different kind of body fluids such as saliva and urine. Most of these DNA fragments are smaller than 100 nucleotides and in order to preserve their integrity, the handling of the sample requires different care. For example, to investigate ultra-short fragments of cfDNA in urine, 10 mmol/L EDTA must be added to the collected urine and stored at -80°C until the DNA fragments are extracted using appropriate protocols and specific resins.

Specifically, using the method involving Wizard resin and guanidine thiocyanate, urine is mixed with guanidine thiocyanate and Wizard Minipreps DNA Purification Resin (Promega). After two hours in constant rotation at room temperature, the mixture is vacuum filtered and finally washed with a solution based on KOAc, Ethanol, TRIS-HCl and EDTA. The DNA will be then eluted with 100µl of nuclease free water at 60°C and later purified [10], [11].

The handling biological fluids require suitable methods and storage times depending on the type of sample and analysts to be examined. The temperature, physical stresses due to sample treatment can lead to chemical and physical changes that may induce sample degradation [12].

**Fig. 1:**
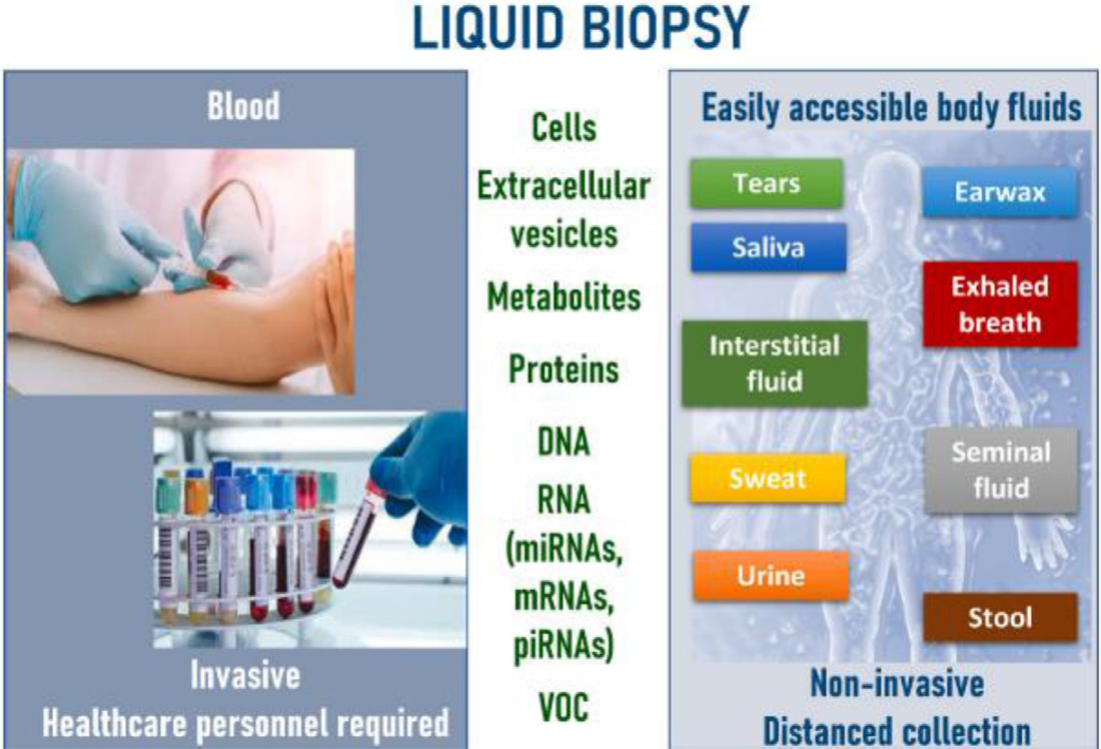
Liquid biopsy from blood and other biological fluid of easy access. Reproduced from [6]

The blood remains the main biological fluid for detecting predictive and diagnostic cancer biomarkers, but also saliva, urine, stool, sweat, breath, seminal fluid and even earwax are samples rich of biological data and easily accessible than blood (Fig. 1). The use of alternative biofluids instead of blood is particularly appealing for the possibility of self-collection (in the most of the cases) which, in pandemic times could avoid or minimize contact with healthcare personnel. Recently, the presence of biomarkers in biological fluids has been investigated in depth and there are several well-established methods for handling, but the translation into common clinical practice is still far from reality and blood remains the gold standard for diagnostic tests [13]. In current analytical procedures indeed, the Prostate Cancer antigen 3 (PCA3) RNA detection of urine is one of the few markers Food and Drug Administration (FDA) approved that can be detected in urine and not in blood for diagnosis and monitoring the disease [14], [15].

In the next pages we will describe the *Saliva, Urine, Stool, Seminal Fluid, Tears and Sweat* handling methods as LB examples above mentioned for the metabolites, nucleic acids, proteins and extracellular vesicles analysis. The main goal of this review is to demonstrate that LB from biological fluid could work as a real alternative to invasive assays for the diagnosis of several diseases which currently require invasive methods like tissue biopsies associated to uncomfortable analysis like colonoscopy for colon cancer or Digital Rectal Examination (DRE) for prostate cancer just to list a few.

## Nucleic acids, metabolites, proteins analysis and Extracellular Vesicles (EVs)/exosomes: sample handling methods

### Saliva

Saliva includes proteins, hormones, antibodies and others molecules that can be also detected usually in the blood [16]. It is produced from salivary glands (parotid, sublingual and submandibular glands) [17]. The main functions of saliva are to assist the food bolus formation through to amylase and other enzymes at an initial digestive activity, to regulate of the immune defense, to protect the mucous membranes and the mineralization of teeth [18].

**Fig. 2:**
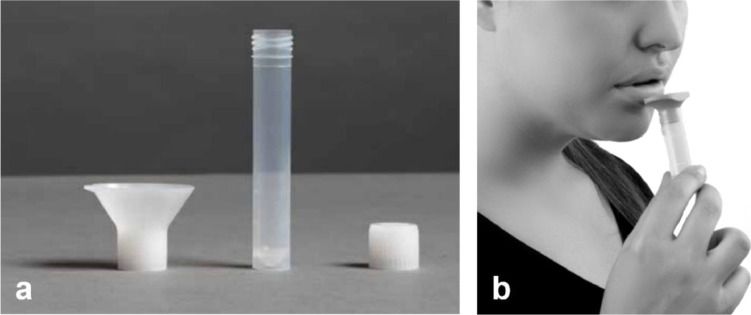
Common kit (a) for simple and not invasive method to self-collect a saliva sample without personnel training(b). Usually, the kit includes a funnel for facilitating sample collection the tube and the tube cap.

The collection and storage of whole saliva samples require a specific procedure that forbid patients from eating and washing mouth at least 1h before sample collection. There are several protocols with common features used to collect saliva. In Fig. 2 is shown a simple method of saliva withdrawal to perform Real Time Polymerase Chain Reaction (PCR) for Severe Acute Respiratory Syndrome Coronavirus 2 (SARS-CoV-2) detection. Among them, some require participants to rinse their mouths for 30 seconds with distilled and deionized water to remove debris and hydrate the mucosa. After 10 minutes, saliva can be collected [19]. Sampling stimulated and non-stimulated saliva depends on the analyte, and it can be collected by passively drooling through lower lip or directly spitting into a vial; using a polystyrene swab; chewing a cotton roll, a polyester or a paraffin wax piece [18]. However, one of the principal drawbacks is the rapid degradation of saliva, which occurs in less than 30 min. The immediate treatment with protease inhibitors together with a 4°C storage may partially prevent a complete degradation, allowing the analysis of sample within the first 4 hours from collection. The recovery of some species like secretory Immune globulin A (s-IgA) or catecholamines can be obtained if the sample is quickly stored at -20°C or mixed with enzyme inhibitors (i.e. leupeptin or aprotinin), glycerol buffers, denaturing agents (trifluoracetate) or sodium azide [20], [21]. If longer storage is required, it is recommended at -80°C.

Using technologies such as Mass Spectrometry, Reverse Transcription Polymerase Chain Reaction (RT-PCR), proteins and nucleic acids like RNA can be detected at lower concentrations in saliva compared to serum [22].

For example, the RNA is extracted from total saliva, including both cell pellet and cell-free supernatants using the QIAzol lysis reagent (Qiagen). The next steps are precipitation using isopropyl alcohol, retrotranscription into complementary DNA (cDNA) and Real Time PCR for gene expression analysis. Sample preservation is critical to avoid or minimize nucleic acids degradation prior to retrotranscription, having consequences in a changing gene expression profile of sample and leading to altered diagnosis and monitoring of disease. To perform the analysis, saliva is collected in 50mL Falcon tubes DNase and RNase free, frozen in dry ice and then stored at -80°C until processed samples. Before to discharge RNA extraction, cells are separated from supernatant trough centrifugation at 4°C for 20 minutes at 11000g [23].

For Oral Cancer, a malignant tumor of the mouth, in patients without relapses and healthy donors, it is possible to carry out metabolic analysis of saliva. Samples are collected and stored immediately at-80°C. Prior to analysis, they are thawed slowly at 4°C, then at room temperature and centrifuged with a 5-kDa cutoff filter. Analyzing several metabolites in saliva, it is demonstrated a clear difference in their concentration between healthy and diseased patients [24].

If protein analysis of saliva is required, before proteins extraction, the samples are collected with a mix of protease inhibitors in previously cooled polypropylene tubes and placed in ice. To remove debris, they are centrifuged at 700*g* for 15 minutes at 4°C. Finally, the collected supernatant is divided into three fractions and stored at -70°C.

Proteins are precipitated using 90% acetone (v/v), 10% TCA (v/v), and 0.07% 2-mercaptoethanol (v/v) and incubated for 24h at -20°C. The next day the samples are centrifuged at 42000*g* for 10 min, at 4°C. The resulting pellet is washed three times with pure acetone containing 2-mercaptoethanol. At the end, the pellet is allowed to air dry and solubilized in 9M urea, 4% 3-((3-cholamidopropyl) dimethylammonium)-1-propanesulphonic acid (w/v), 0.05% Triton X100 (v/v), and 65mM dithiothreitol (DTT). Before continuing with the protein analysis, performing One-Dimension, Two-Dimension Electrophoresis and Mass Spectrometry techniques, the protein assay is carried out with the Bradford method [25].

EVs and in particular microvesicles and exosomes are other biomarker categories. Even if they are very difficult to investigate and there is still a lack of information on their role and composition as to date they are on the rise as early biomarkers. Right now, there are no standardized methods to distinguish between different types of EVs as the proteomic profiles of particles change depending on the isolation method used, even if they come from the same cell line [26]. According to one of available protocols to isolate exosomes from whole saliva, samples are collected in sterile tubes and immediately subjected to centrifugation at 3000*g* for 20 min at 4°C as a necessary step to deplete cells and debris. The supernatant is preserved at -70°C until sample handling. The exosomes isolation can be achieved employing two various methods: using chemical agents such as ExoQuick-TC™ kit, containing a patented polymer used to precipitate exosomes and microvesicles falling in the size range from 30 to 200nm [27] and physical methods based on ultracentrifugation [28].

### Urine

Urine is a bodily fluid in which over 3000 metabolites has been discovered in recent decades. Today, urine components are considered biomarkers for diagnosis of several diseases. Each person produces about 1,5-2 liters of urine per day. The kidneys continuously filter, secrete and reabsorb various substances from all over the body, producing urine that include metabolites, nucleic acids, protein and exosomes.

This biological waste fluid contains high concentrations of urea (obtained by the metabolism of amino acids), chloride, sodium, potassium, creatinine, ammonia, organic acids, various water-soluble toxins and urobilin, which gives it the characteristic color. Its composition changes according to various factors such as gender, age, exercise and food [29]. Urine is among the most easily accessible biological samples and can be collected in large volumes because its withdrawal is non-invasive. It has a less complex composition than blood and its features allow diagnosis and screening of diseases such as cancer [30].

Usually, metabolites in urine are investigated using Nuclear Magnetic Resonance (NMR) Spectroscopy, Gas Chromatography Mass Spectrometry (GC-MS), Direct Flow Injection Mass Spectrometry (DFI/LC-MS/MS), Inductively Paired Plasma Mass Spectrometry (ICP-MS) and High-Performance Liquid Chromatography (HPLC) experiments performed. With this purpose, the samples collected are treated with sodium azide, centrifuged at 4000*rpm* for 10 min to move away particulate. The urine samples are frozen in falcon tubes at -20°C up to use. Before every analysis, samples defrost at room temperature for 30 minutes, filtered and centrifuged [31].

The urine is also a source of nucleic acids that can give information about the health of the patient. As a general rule, the samples are collected, centrifuged and stored at -80°C (storage in this condition is allowed for a long time). To carry out RNA extraction, the urine is defrosted and divided into 100μl aliquots, 700μl of Qiazol reagent (Qiagen) are added to each and frozen until the RNA will be extracted, measured out spectrophotometrically and reverse transcribed into cDNA for the next analysis [32]. In the keeping urine samples, the storage temperature is a determining factor in preventing the biological molecules degradation in the urine. In particular, it allows protein, potassium, amylase integrity and bacterial growth remain unaffected, unlike concentrations of sodium, urea, albumin, creatinine and uric acid, which remain stable up to 72 hours after collection regardless of storage temperature [33].

To perform the proteomics study of biomarkers in urine, the sample handling method the protocol steps require that the sample is collected in a sterile container and transported on ice to prevent microbial contamination and proteolysis. Insoluble components are removed by centrifugation at 2000*g* at 4°C for 10 minutes within 30 minutes of collection to prevent the release artifacts. The supernatant is then collected and stored at -20°C for short times and at -80°C for longer times. Urine samples are examined for color, turbidity and pH before processing to prevent alterations [34].

Over the last years, the number of identifying proteins has increased thanks to new approach introduced for the analysis. So, combining one-dimensional gel electrophoresis and reverse phase liquid chromatography coupled to Mass Spectrometry, it is possible to identify more than 2000 proteins in a urine sample from a normal individual. In order to study the urinary proteome, it is necessary to desalinate the sample through the process of precipitation, lyophilization, ultracentrifugation or centrifugal filtration. Then, depending on the technique used, the sample will be processed differently and analyzed by one of the listed methods: 2-Dimensional gel Electrophoresis followed by Mass Spectrometry (2DE-MS), Liquid Chromatography combined with Mass Spectrometry (LC-MS), Surface-Enhanced Laser Desorption/Ionization combined with Mass Spectrometry (SELDI-TOF), and Capillary Electrophoresis combined with Mass Spectrometry (CE-MS) and protein microarrays [35].

Moreover, urine is liquid rich in circulating miRNAs and miRNAs enclosed secreted extracellular vesicles. The latter is protected from degradation processes. RNA is thus preserved from urine passage through the last tract of the excretory system. The EVs are a heterogeneous population of membranous bodies and have a diameter between 40 and 200nm, while the micro-vesicles are within 50-1000nm. Exosomes are isolated from the total urine by a process of serial centrifugation conducted at 4°C. They are collected from 50mL of morning-urine in sterile plastic tubes. The first centrifugation is done at 200*g* for 20 min to remove the cellular components, the second at 2000*g* for 20 min to separate debris, bacteria and other proteins. A third centrifugation at 16000*g* for 20 min will eliminate ectosomes (plasma membranes) [36].

### Stool

Feces are a waste product consisting of water, bacteria, food components and metabolites. Due to a close correlation between stool and gut, changes in the intestine are reflected in the feces and its metabolic composition. For these reasons, metabolic analysis could contribute to the early diagnosis of Inflammatory bowel diseases (IBD) and Colorectal cancer (CRC) avoiding colonoscopy. Hundreds of different metabolites (amino acids, lipids, triglycerides) have been discovered in stool and can be extracted using some solvents such as isopropanol, ethanol, methanol or a combination of these depending on analyte kind to be extracted.

Currently, the CRC accounts for 10% of fatal cancers. The incidence has increased over years due to an unhealthy lifestyle, including little attention to food, sedentary life and neglected early screening tests [37].

Like CRC, the diagnosis of IBD, such as Crohn's disease and ulcerative colitis occurs with the detection of occult blood in feces [38] that is usually a prelude to an invasive examination such as colonoscopy. Thus, the development of sensitive and specific tests enabling a diagnosis without recurring to more costly and painful tests could be of interest for medical societies and patients. Some fecal tests used for bowel disease screening are available, performing a reaction with the hemoglobin heme group. Other tests, specific for CRC diagnosis are based on immunochemical tests to detect the presence of human hemoglobin and are sensitive for adenomas and advanced neoplasm diagnosis. Instead, the combination of immunochemical exams and multiple DNA markers has ensured progress in the diagnosis and discrimination among all these diseases and their progression stage [39].

**Fig. 3:**
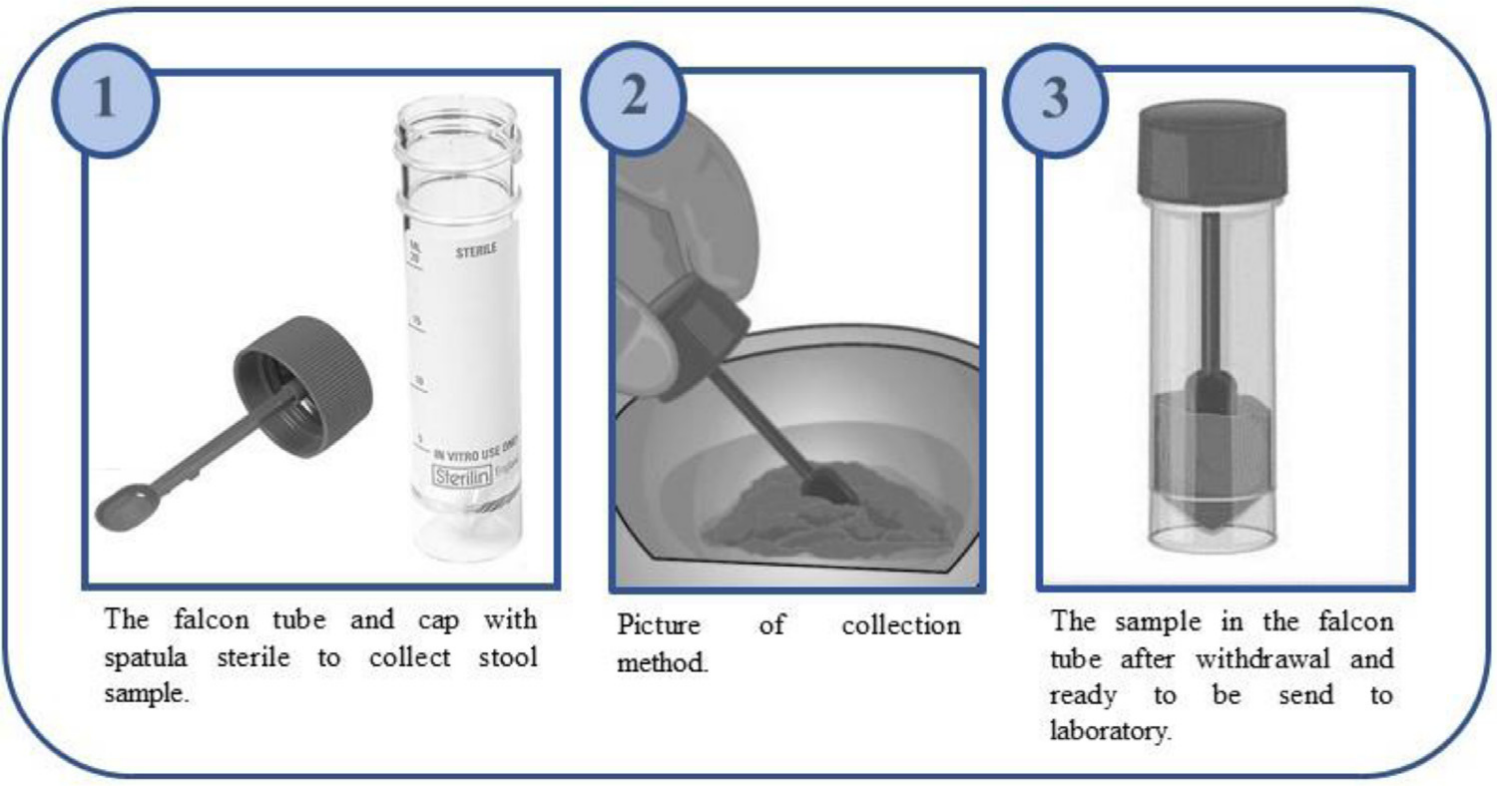
Stool sample collection and transport method.

In recent decades, it has been shown that microbiota imbalance can be linked also to diseases such as obesity, type 2 diabetes and cardiovascular diseases. Several protocols can be used to the proteomics microbiota assays that needed protease digestion and desalination, however, extracting proteins from microorganisms is complex due to differences in cell structures such as the cell wall. In all cases, the starting point is the collection of feces (Fig. 3), which are immediately placed on ice, transferred to the laboratory.

For the purpose of extraction microbial DNA from feces, some protocols are available in the literature. Because fecal samples are rich in microbes, especially bacteria, nucleic acid extraction methods focus on the latter with the ultimate goal of sequencing the 16S rRNA gene or the whole genome [40].

In detail, for metabolomics measurements, the samples are collected from patients in tubes with a special small spoon and frozen at -20°C at home. They are transported to insulate and refrigerated environment and refrozen in the laboratory at -20°C for one week and then at -80°C (also for months) before analysis [41].

Collected fecal specimens, if processed within 20 minutes, can be instead temporarily kept at room temperature. If the sample is destined to DNA extraction has to be shipped, storage on dry ice is required otherwise, a commercial brand stabilizer (DNA Genotek P-084 for DNA extraction, DNA Genotek P-085 for DNA and RNA extraction, Stratec PSP Spin stool DNA plus kit, brown Sarstedt) may be added to the sample after collection, mixed vigorously and shipped at room temperature. As a matter of fact, stabilizers are DNase inactivates preventing degradation of DNA and preserving microorganism titer. They allow the extracted DNA molecules to be preserved for a long-term room temperature (up to three months) [42].

A standard protocol for feces analysis includes several steps of centrifugation, starting with about 2 g of stool, resuspended in 10mL of cold PBS and vortexes in the presence of glass beads. First centrifugation step is at 300*g*, 4°C for 5 min to retain the supernatant. The pellet is processed to other centrifugations and the supernatants set aside are further subjected to three centrifugations at 300*g*, 4°C for 5 min to remove debris and larger particles. The resulting product was centrifuged at 14000*g*, 4°C for 20 min. The pellet obtained is washed three times with cold PBS and centrifuged at 14000*g*, 4°C again for 20 min. At this point, the protein extraction from the obtained microbial cells can be accomplished [43].

The human microbiota includes trillions of microbes from both gram-negative and gram-positive bacteria. As shown by Eun-Young Lee and colleagues, gram-negative bacteria secretory outer membrane vesicles (OMVs) in the extracellular environment that may contribute to the onset of intestinal cancer. The EVs production by both human cells and bacteria suggests that the secretion of membrane vesicles is an evolutionarily conserved process [44]. Also, for this purpose, the sample is self-collected and stored in the same way as described above. After sample thawing, to prepare samples for EVs extraction, samples are filtered through a cellular filter after being diluted in 10mL of PBS for 24h [45].

### Seminal Fluid

Seminal Fluid (SF) or also called Semen are source rich of biomarkers that can use to diagnose male infertility and reproduction disorders. Infertility affects about 15-20% of couples worldwide. In addition to disorders of the male reproductive system, prostate cancer is one of the diseases that can be diagnosed through seminal fluid analysis [46].

Each man produces around 4mL of seminal fluid containing 600 million spermatozoa for each ejaculation, where the main component is seminal plasma. SF, like blood, is one of the biological samples richer in proteins (it contains over 10000 different proteins). Ejaculate includes different types of constituents: there are epithelial and myeloid cells originating from the male urogenital tract, but also amino acids, proteins, sugars and metabolites. Among these, fructose promotes sperm viability in a hostile environment such as the female reproductive system [47]. From a metabolic point of view, fructose, myo-inositol, aspartate and choline can influence the total number of spermatozoa (oligozoospermia) and their motility.

Oligospermia is a pathological condition in which a sample of ejaculate shows fewer than 15 million spermatozoa/mL, sperm motility is less than 40% and the number of normal sperms is less than 4%.

After a 3-5day period of sexual abstinence, sperm samples achieved through masturbation are incubated for 30 minutes at 37°C and allowed to liquefy. To eliminate the cellular component, samples are centrifuged twice at 4°C. The first centrifugation is performed at 1600*g* for 10 minutes at 4°C, the supernatant collected is centrifuged again at 16000*g* for 10 minutes and cells and cellular debris are removed. The supernatant is then collected and used for subsequent investigations [48].

To Nuclear Magnetic Resonance (NMR) assay, in example, the sample should be treated with methanol, milli-Q water and chloroform, vortexed for 1 min and centrifuged at 4500*rpm*, 15 min at 4°C. The supernatant obtained, which contains low-weight molecules, can be also analyzed throughout NMR spectroscopy in order to identify the metabolites available in the SF [49].

Lately, it was discovered that seminal plasma in a large number of biomarkers, contains a lot of cell-free double stranded RNAs, long single stranded RNA, small RNAs, miRNA and piwi-interacting RNA (piRNA). Indeed, the nucleic acids can phenotypically influence the zygote development and consequently the offspring [50]. Human SF includes also a lot of Seminal Micro-Vesicles (SMVs) produced by the prostate and epididymis into the lumen of the gland and released through an apocrine secretion.

To carry out nucleic acids’ isolation, the semen is centrifuged at low speed to avoid cell lysis. In detail, an initial centrifugation at 400g for 10 minutes and then at 2000*g* for 20 min is done. Alternatively, it may be centrifuged at 16000*g* for 5 min. The supernatant achieved after low speed centrifugation is processed in two ways before going further with nucleic acid isolation. It may be sieved through filters with a pore size of 0,22µm or centrifuged at 16000*g* for 5 minutes after incubation at 37°C for 0, 6 and 24h [51].

Seminal Fluid Proteins (SFPs) are involved in reproductive activity influencing fertilization and fertility by driving egg-sperm cell interaction [52]. They play several key roles such as sperm protection and maturation. They modulate the immune response in male and female reproductive tracts, ensuring that spermatozoa arrive and recognize the oocyte [53].

To allow protein analysis, protease inhibitors are immediately added to the collected sample. To this aim, it is centrifuged at 800*g* for 15 min (4°C) and the supernatant is transferred to sterile tubes. A second centrifugation at 10000*g* for 30 min (4°C) is done. The supernatant is stored in sterile tubes at -80°C until employment, such as Mass Spectrometry and liquid chromatography-mass spectrometry [54].

To avoid degradation of biomarkers in seminal fluid, the sample must be brought to the laboratory within 2 hours from collection. But if the objective is to analyze sperm motility, it is appropriate within 1h. If the sample is intended for use in Assisted Reproductive Technologies (ART), it must be frozen in liquid nitrogen at -196°C to preserve sperm motility, DNA integrity and to ensure the cells' ability to fertilize. Ascorbic acid, resveratrol, dimethylsulfoxide, antioxidants (β-mercaptoethanol) and glycerol supplementation can be used as preservative agents in culture media [55].

### Tears

Tear fluid (TF) is produced by the nasolacrimal duct and the lacrimal glands. It has multiple functions such as protecting the eyes against infection, lubricating the eyelids, conjunctiva and cornea; Tears help to remove foreign material from the eyes, moreover, they maintain the microenvironment of the ocular surface and changes in their quality and quantity can lead to dry eyes [56] and others eye diseases.

This body fluid is characterized lipids, carbohydrates, salts and proteins, including mucin, lysozyme, lactoferrin, and secretory immunoglobulin A, that are antimicrobial factors with protective role [57].

Another protein, the lipocalin, is contained inside of tears and binding cholesterol, fatty acids, fatty alcohols, glycolipids, phospholipids and others lipids, allowing them to increase their solubility in aqueous solution [58].

To perform more analysis, tear samples are collected using a Schirmer's strip [59]. The Schirmer's test requires the use of a tear strip inserted into the lower ocular fornix without anesthesia and removed after 5 minutes. The length of the wetted area is then assessed. The strip can be stored at -80°C until resulting analysis [60].

TF is rich in metabolites that can be typified giving information about ocular surface disorders. Through the Schirmer's method, 5µl /10µl of sample volume is collected. Furthermore, employing techniques such as Nuclear Magnetic Resonance (NMR), Gas chromatography–mass spectrometry (GC-MS) and Liquid chromatography–mass spectrometry (LC-MS), it is possible to analyse fewer metabolites identified than in other biological fluids to date [61].

An eye disorder that can be studied thanks to LB of tears is the Thyroid eye disease (TED). It is a pathology resulting from autoimmune thyroid disease. It occurs as a result of enlarged extraocular muscles, fatty tissue and connective tissue. Over time it can lead to exophthalmos, limitation of the eye muscles and in severe cases, blindness as a result of corneal rupture or compressive optic neuropathy. Also, dry eye disease (DED) is a pathology involving tear composition that causes visual disturbances and variability of the tear film, provoking damage to the ocular surface.

To collect the samples, 5µl of tear fluid is ran up from the lower eyelid tip and lower fornix through a blunt glass micro-capillary tube. The samples collected in DNase-free microtubes and added to the PicoGreen dye within 4 hours of withdrawal. Once the sampling procedure is achieved, the tubes are kept on ice [62].

In patients TF, interleukins (IL)-1β, IL-6, IL-13, IL-17A, IL-18 and tumor necrosis factor (TNF-α) are increased compared to healthy subjects. Cytokines perform their functions in soluble form, associated with exosomes or encapsulated in the exosomes. Indeed, tears of TED patients contain very high concentrations of exosomes, indicating that they may be involved in the pathogenesis of ophthalmic diseases. This evidence suggests that tear exosomes may be biomarkers of disease that can be collected non-invasively.

To preserve integrity of TF for further exosomes analysis, a polyvinyl acetal eye sponge on the inferior conjunctival sac is used, recurring to general anesthesia for the patient. The sponge is placed in a 0.2 ml tube which is centrifuged at 13,000*rpm* at 4°C for 30 min. The TF obtained is stored at -80°C. Exosomes are then isolated using Exosome Isolation Reagent. The recovered pellet is resuspended in phosphate-buffered saline and quantification is carried out using a bicinchoninic acid (BCA) assay [63].

Among the eye diseases Retinoblastoma is the most common intraocular tumor in children. Also, in this case, liquid biopsy allows monitoring of therapeutic response by investigation of tumor-derived cell-free DNA (cfDNA) in the aqueous humor (AH) a fluid which can be collected by paracentesis of the clear cornea.

Unlike other biological fluids, AH can be collected invasively, but a few microliters may be representative of the entire biological fluid allowing cancer follow-up [64].

### Sweat

The sweat is a body fluid secreted from sweat glands onto human skin surface. Its main role is the thermoregulation of human body but sweat glands trough sweating, allow to remove waste products and toxic substances in the same manner as other excretory organs such as the kidneys and intestines, albeit to a lesser extent [65].

The most of biomolecules contained in this body fluid are electrolytes, amino acids, metabolites, hormones, proteins, nucleic acids, micronutrients [66], carbohydrates, lactate, urea, volatile organic compounds (VOCs) and sebum components excreted by the body as they are able to provide information about the physiological state of the organism [67].

The sweat glands are divided into eccrine, apocrine and sebaceous; each day they produce between 300 and 700 ml of sweat throughout the body and up to 2 to 4 liters per hour during strenuous exercise [68].

There is a correlation between blood plasma and the sweat composition of certain lipophilic biomolecules (steroid hormones and drugs) as they transport across lipophilic cell membranes. Sweat can therefore be analyzed to assay the concentration of drugs, for the study of new therapies with a personalized approach. It is also rich in miRNAs conveyed by extracellular vesicles. Sira Karvinen et al. showed that when a group of people exercise at different intensities, sweat is enriched in specific miRNAs sequences related to the exercise performed and not simply on an increase in body temperature [69].

Performing a proteomic and metabolomic analysis of the same sweat samples, it was found that the main groups of proteins have hydrolase and catalytic activity, while amino acids remain the most abundant metabolites. Catalytic activity leads to an increased release of free amino acids in sweat, whose levels vary with exercise [70].

For each type of analysis, there are different ways of collecting sweat. The subject is asked to walk for 30 minutes in an environment heated to 35°C temperature and fresh sweat is collected through a glass Pasteur pipette with a latex bulb. It is transferred Eppendorf micro-tubes and stored at -80°C until sample preparation. The sweat, often, cannot be analyzed after withdraw because of logistical reasons. On one hand, the storage temperature does not affect sodium, chloride, potassium and urea concentrations, but on the other to preserve lactate and ammonia concentrations in sweat, the sample should be stored at room temperature to be used before 7 days; if longer storage (until 28 days) is required, it should be kept at -20°C [71].

Another way to collect sweat is to use gauze or ashless filter paper which is previously wetted with ethanol, phosphate buffer solution pH 7 or a mixture of both. It will be rubbed onto the affected skin area and stored in 50 ml falcon plastic tubing at -80°C until the sample is prepared [72].

## Conclusion and future perspective

The LB has the advantage of ensuring large-scale screening of diseases, in particular in the cancer field. The reasons why LB is becoming increasingly relevant in clinical practice are due to its main features such as being non-invasive, rapid, accurate, easily repeatable and low-risk [73] compared to tissue biopsy. It has several application perspectives in diagnosis, treatment and monitoring in real-time and follow up of diseases but also in early detection of cancer and drug resistance. With these assumptions, it could overcome the limitation of tumor heterogeneity and replace tissue biopsy in the future [74]. Moreover, in the last two years of COVID-19 pandemic limitations, LB from easily accessible biological fluids could represent an interesting tool in facilitating screening, diagnosis and follow-up of diseases without the intervention of specialized personnel. In this paper the potential that each easy-access biological fluid (saliva, urine, stool, seminal fluid) has in “distanced diagnostics” is considered and protocols able to preserve diagnostic elements are provided. In Table 1 are summarized biomarkers and handling methods for each sample type abovementioned.

**Table 1:** Biomarkers from saliva, urine, stool, seminal fluid and handling methods.

Type of samples	Type of biomarker	Sample handling methods
Saliva	Metabolites	Collect and store immediately at -80°C. Prior to analysis, samples are thawed slowly at 4°C, then at room temperature and centrifuged with a 5-kDa cutoff filter.w
	Nucleic acids	Collect in 50mL Falcon tubes DNase and RNase free, frozen in dry ice and then store at -80°C until processes. Cells are separated from supernatant trough centrifugation at 4°C for 20 minutes at 11000g.
	Proteins	Collect and add a mix of protease inhibitors in previously cooled polypropylene tubes and place in ice. To remove debris, centrifuge at 700g for 15 minutes at 4°C. Finally, store the collected supernatant at -70°C.
	Vesicle/exosomes	Collect in sterile tubes and immediately centrifuge at 3000*g* for 20 minutes at 4°C, for depletion of cells and debris. The supernatant is preserved at -70°C until sample handling.
Urine	Metabolites	Collect and treat with sodium azide, centrifuge at 4000*rpm* for 10 minutes to move away particulate. Freeze urine samples in falcon tubes at -20°C up to use. Before every analysis, thaw samples at room temperature for 30 minutes, filter and centrifuge.
	Nucleic acids	Collect, centrifuge and store -80°C.
	Proteins	Collect in sterile containers and transport on ice to prevent microbial contamination and proteolysis. Remove insoluble components by centrifugation at 2000*g* at 4°C for 10 minutes within 30 minutes of collection. Store at -20°C for short times and at -80°C for longer times.
	Vesicle/exosomes	Collect 50mL of morning-urine in sterile plastic tubes and perform serial centrifugation at 4°C. First centrifugation at 200*g* for 20 minutes to remove the cellular component; the second one at 2000*g* for 20 minutes to move away debris, bacteria and other proteins. A third centrifugation at 16000*g* for 20 minutes will separate ectosomes.
Stool	Metabolites	Self-collection sample tubes with a special small spoon and freeze at -20°C at home. Transport to an insulated and refrigerated environment and refreeze in the laboratory, at -20°C for one week and then at -80°C before bioassays.
	Nucleic acids	Maintain sample at room temperature if processed within 20 minutes otherwise store at -20°C for short periods and at -80°C for longer time. Alternatively, add a stabilizer to the sample after collection, mix vigorously and ship at room temperature.
	Proteins	Resuspend 2g of stool in 10mL of cold PBS, vortex in the presence of glass beads and centrifuge at 300*g*, 4°C for 5 min to retain the supernatant. Centrifuge supernatants for another three times at 300*g*.
	Extracellular Vesicles (EVs)	Self-collect and freeze -20°C, transport to the laboratory and freeze at -70°C.To prepare samples for EVs extraction, thaw and filter samples through a cellular filter after being diluted in 10mL of PBS for 24h.
Seminal fluid	Metabolites	Collect and centrifuge for 10 minutes at 12000*g* at 4°C and the resulting supernatant is ready for metabolic analysis.
	Nucleic acids	Collect and centrifuge at low speed to avoid cell lysis. Initial centrifugation at 400*g* for 10 minutes and then at 2000*g* for 20 min. Alternatively, centrifuge at 16000*g* for 5 minutes.
	Proteins	Collect and add protease inhibitors to sample. Centrifuge at 800*g* for 15 minutes (4°C) and transfer the obtained supernatant into sterile tubes. Do a second centrifugation at 10000*g* for 30 minutes (4°C). Store the supernatant in sterile tubes at -80°C until analysis.
	Seminal Micro-Vesicles (SMVs)	Collect and incubate for 30 minutes at 37°C and allow to liquefy. Eliminate the cellular component: centrifuge samples twice at 4°C. Perform the first centrifugation at 1600*g* for 10 minutes at 4°C, collect supernatant and centrifuge it again at 16000*g* for 10 minutes and cells and cellular debris are removed.

Others body fluids

Scientific progress in biomarker identification in several biological fluids indeed, has opened new perspectives in the diagnostic field, enabling self-collection of samples without the pain and embarrassment of patients. On the other hand, self-collection of the sample depends on its stability and way it is stored and transported for analysis, in order to preserve elements that are biological fluid and have to be analyzed.

But the use of biomarkers of biofluids alternative to blood the diagnosis and monitoring is currently limited to the field of research. These limitations are caused by a high false-negative rate, requiring a confirmatory tissue biopsy. This is often related to the heterogeneity of diseases, especially cancer [75].

The LB with its biomarkers has the potential to be a tool for the diagnosis, prognosis and evaluation of a patient with diseases such as cancer. To put in practice this potential, it will be appropriate to validate and standardize every step of the workflow such as sample collection, preservation, transfer, storage, analysis, data management and analysis including data interpretation [76].

The purpose of diagnostic or screening tests depends on its validity, considering its sensitivity and specificity [77]. Even a small fault of test specificity could bring to false positive results, provoking worry patient concern and enormous costs for subsequent diagnosis. False-negative results, on the other hand, could set back timely and important therapeutic intervention [78]. Another big issue for liquid biopsy is the need to preserve the integrity of biological species contained in the sample, to allow LB being a 'snapshot' of a pathophysiological condition. There are many steps to be considered to ensure the sensitivity of early diagnostic tests. To this end, the proper collection and storage of the biological sample plays a very important role. To bridge this gap, translational clinical trials will establish the diagnostic accuracy and clinical utility of biomarkers of LB, which should be able to ensure the appropriate adherence to international rules of standardization and validation setup by International control institutions like the FDA, CEN-CENELEC and ETSI^1^. In the future, LB will need to show sensitivity, specificity, accuracy and reproducibility to be accepted in clinical trials and subsequently gain approval. The goal within the next 10 years is to control cancer and its metastases through the use of LB, thus monitoring disease progression and providing information to physicians on therapeutic approaches [79].

## Declaration of Competing Interest

The authors declare that they have no known competing financial interests or personal relationships that could have appeared to influence the work reported in this paper.

## Data Availability

Data will be made available on request.

## Footnotes

1 CEN-CENELEC organization (https://www.cencenelec.eu/) and ETSI organization (https://www.etsi.org/)
